# Transcriptional landscape of myasthenia gravis revealed by weighted gene coexpression network analysis

**DOI:** 10.3389/fgene.2023.1106359

**Published:** 2023-03-27

**Authors:** Demin Zhang, Liqin Luo, Feng Lu, Bo Li, Xiaoyun Lai

**Affiliations:** Department of Neurology, The 923rd Hospital of the Joint Logistics Support Force of the People’s Liberation Army, Nanning, China

**Keywords:** myasthenia gravis, biomarkers, WGCNA, infiltrated immune cells, LASSO

## Abstract

**Background:** As one of the most common autoimmune diseases, myasthenia gravis (MG) severely affects the quality of life of patients. Therefore, exploring the role of dysregulated genes between MG and healthy controls in the diagnosis of MG is beneficial to reveal new and promising diagnostic biomarkers and clinical therapeutic targets.

**Methods:** The GSE85452 dataset was downloaded from the Gene Expression Omnibus (GEO) database and differential gene expression analysis was performed on MG and healthy control samples to identify differentially expressed genes (DEGs). The functions and pathways involved in DEGs were also explored by functional enrichment analysis. Significantly associated modular genes were identified by weighted gene co-expression network analysis (WGCNA), and MG dysregulated gene co-expression modular-based diagnostic models were constructed by gene set variance analysis (GSVA) and least absolute shrinkage and selection operator (LASSO). In addition, the effect of model genes on tumor immune infiltrating cells was assessed by CIBERSORT. Finally, the upstream regulators of MG dysregulated gene co-expression module were obtained by Pivot analysis.

**Results:** The green module with high diagnostic performance was identified by GSVA and WGCNA. The LASSO model obtained NAPB, C5orf25 and ERICH1 genes had excellent diagnostic performance for MG. Immune cell infiltration results showed a significant negative correlation between green module scores and infiltration abundance of Macrophages M2 cells.

**Conclusion:** In this study, a diagnostic model based on the co-expression module of MG dysregulated genes was constructed, which has good diagnostic performance and contributes to the diagnosis of MG.

## Introduction

As an autoimmune disease, myasthenia gravis (MG) manifests primarily as fluctuating muscle weakness caused by autoantibodies and cell-mediated disruption of acetylcholine receptors (Nations et al., 1999). It is characterized by dysfunctional transmission of the neuromuscular junction, resulting in muscle weakness (Meriggioli and Sanders, 2009). MG reduces the quality of life of patients and can be life-threatening in severe cases (Phillips, 2004; Andersen et al., 2014; Bettini et al., 2017). The prevalence of MG is estimated to be 0.3–2.8/100,000, with a global prevalence of 700,000, and the current mortality rate of MG is 5%–9% (Alshekhlee et al., 2009; Carr et al., 2010; Deenen et al., 2015). In recent years, many advances have been made in the treatment of MG, and more evidence-based medical evidence has been accumulated, which has significantly improved the prognosis of the vast majority of patients and enabled the effective control of a small number of refractory MG cases (Batocchi et al., 2000; Vincent and Drachman, 2002; Rowin et al., 2004; Grob et al., 2008; Muscle Study, 2008). However, the clinical manifestations of MG are highly heterogeneous (Rodolico et al., 2002; Grob et al., 2008). Identifying potential biomarkers of MG will help in the diagnosis and treatment of MG.

Currently, serological tests for autoantibodies are commonly used for the diagnosis and disease classification of MG patients (Gilhus and Verschuuren, 2015). About 85% of MG patients have antibodies against the muscle acetylcholine receptor (AChR) (Higuchi et al., 2011). In addition, antibodies against muscle-specific kinase (MuSK) were found in about 6% of patients (Berrih-Aknin et al., 2014), and antibodies against LRP4 were found in about 2% of MG patients (Berrih-Aknin et al., 2014). The pathogenicity of all these autoantibodies has been demonstrated by animal studies (Mori et al., 2012; Shen et al., 2013). However, the pathogenicity of these disease biomarkers is usually uncertain. There is still a need to identify new biomarkers to complement existing diagnostic tools.

Therefore, in this study, we identified dysregulated genes in MG patients and performed a weighted gene co-expression network analysis (WGCNA) on these genes. In addition, we further developed a clinical diagnostic model based on dysregulated genes and revealed the relationship between this clinical diagnostic model and the multi-omics landscape of immunological features and global regulatory networks.

## Materials and methods

### Data resources

In this study, the MG dataset GSE85452 (Mamrut et al., 2017) was downloaded from the Gene Expression Omnibus (GEO) database (http://www.ncbi.nlm.nih.gov/geo/). The GSE85452 dataset is based on the GPL10558 platform and contains the mRNA expression profiles of 13 MG and 12 healthy control PBMCs.

### Differential gene expression analysis

To identify differentially expressed genes (DEGs) between control and MG samples, differential gene expression analysis was performed using the Bioinforcloud application DEbylimma, which was developed based on the limma package (Ritchie et al., 2015). Among the differences, those associated with *p* < 0.01 and |logFC|> 0 were considered significant. Subsequently, heat maps were drawn using the Bioinforcloud application PlotHeatmap to further demonstrate the expression of DEGs between samples.

### Weighted gene co-expression network analysis

The weighted gene co-expression network analysis (WGCNA) application in Bioinforcloud was based on the WGCNA package in the R language (Langfelder and Horvath, 2008) being used to perform WGCNA on DEGs. Candidate powers (Nations et al., 1999; Batocchi et al., 2000; Rodolico et al., 2002; Vincent and Drachman, 2002; Phillips, 2004; Rowin et al., 2004; Grob et al., 2008; Muscle Study, 2008; Alshekhlee et al., 2009; Meriggioli and Sanders, 2009; Carr et al., 2010; Higuchi et al., 2011; Mori et al., 2012; Shen et al., 2013; Andersen et al., 2014; Berrih-Aknin et al., 2014; Deenen et al., 2015; Gilhus and Verschuuren, 2015; Bettini et al., 2017; Mamrut et al., 2017) were used to test the average connectivity of the different modules and their independence. Powers were selected if the degree of independence was >0.85. The samples were clustered by using the hclust function of the WGCNA package and checked for outliers. Subsequently, a heat map of module-phenotype correlations was constructed to find module-phenotype correlations and their significance. A high correlation means that the genes of the corresponding module also tend to be highly correlated with the disease state.

### Functional enrichment analysis

Gene Ontology (GO) and Kyoto Encyclopedia of Genes and Genomes (KEGG) enrichment analysis of MG dysregulated gene co-expression module genes using the Bioinforcloud application RunMutiGroupclusterProfiler. The application was developed based on the clusterProfiler package in the R language (Yu et al., 2012), and the enriched functions or pathways were considered significant when *p*< was 0.05.

### Gene set enrichment analysis

Gene set enrichment analysis (GSEA) was performed using the Bioinforcloud application RunGSEA to further explore the potential biological properties of MG dysregulated gene co-expression modules. The application uses the Molecular Signature Database (MsigDB) (Liberzon et al., 2015) of c2. cp.kegg.v7.0. symbols.gmt as the reference gene set, and was developed based on the clusterProfiler package in the R language (Yu et al., 2012), the enrichment results at *p* < 0.05 were considered significant.

### Gene set variation analysis

Gene set variation analysis (GSVA) of modular genes using RunGSVA. The application is based on the GSVA package (Hanzelmann et al., 2013) for calculating GSVA scores of MG dysregulated gene co-expression module genes in different samples. Subsequently, heat maps were drawn using the Bioinforcloud application PlotHeatmap to further demonstrate the expression of GSVA scores across samples.

### Assessment of diagnostic efficacy

Evaluation of diagnostic efficacy of potential markers using the Bioinforcloud application PlotROC. The application is based on the pROC package in R (Robin et al., 2011) and the results were plotted as receptor operating characteristic (ROC) curves. In this study, the potential of GSVA scores of MG dysregulated gene co-expression module genes as a diagnostic marker for MG was evaluated using this application. In the case of area under the curve (AUC) > 0.5, the closer the AUC is to 1, the better the diagnosis.

### Construction of minimum absolute shrinkage and selection operator models

The least absolute shrinkage and selection operator (LASSO) has a strong predictive value and low correlation and is suitable for selecting the best features for high-dimensional data. LASSO regression analysis was performed using the Bioinforcloud application RunLASSO, which was developed based on the glmnet software package (Friedman et al., 2010) and extracted the expression profiles of MG dysregulated genes and co-expression module functional genes with diagnostic efficacy to construct the LASSO model. The expression values of the selected genes were weighted using the regression coefficients of the LASSO analysis to create a model index for each sample with the following equation: Index = ExpGene1*Coef1 + ExpGene2*Coef2 + ExpGene3*Coef3 +.

“Coef” is the regression coefficient of the gene, derived from LASSO Cox regression, and “Exp” indicates the expression value of the gene, thus constructing the MG dysregulated gene co-expression module-based Lasso model.

### Immune cell infiltration analysis

In this study, immune cell infiltration analysis was performed using the Bioinforcloud application RunCIBERSORT to assess the abundance of immune cell infiltration in MG as well as between control samples. The application is based on the CIBERSORT tool (Chen et al., 2018). It was developed to enable the estimation of immune infiltration for large volumes of transcripts and thus assess the relationship between gene expression or other phenotypes and immune cell infiltration. In addition, correlation analysis was performed using the Bioinforcloud application PlotCor to explore the correlation between MG dysregulated gene co-expression module-based models, model genes and the abundance of immune cell infiltration, immune checkpoint genes and tertiary lymphoid structural marker genes.

### Identification of upstream regulators

In this study, the differentially expressed RNA binding proteins (RBPs) were screened in combination with the results of differential gene expression analysis, and subsequently, the upstream regulators regulating the gene sets of the MG dysregulated gene co-expression module-based model were identified using the Bioinforcloud application Pivot. The application is based on a hypergeometric approach to implement Pivot analysis to identify RBPs in the regulatory gene set.

### Data analysis and statistics

All statistical analyses were performed in the Bioinforcloud platform (http://www.bioinforcloud.org.cn), which was applied by calling the appropriate R package. Comparisons between the two groups were made using Student’s t-test and correlation coefficients were calculated using Spearman analysis. *p* < 0.05 was considered significant.

## Results

### Dysregulated gene co-expression modules characterize the global regulatory pattern of myasthenia gravis

**FIGURE 1:**
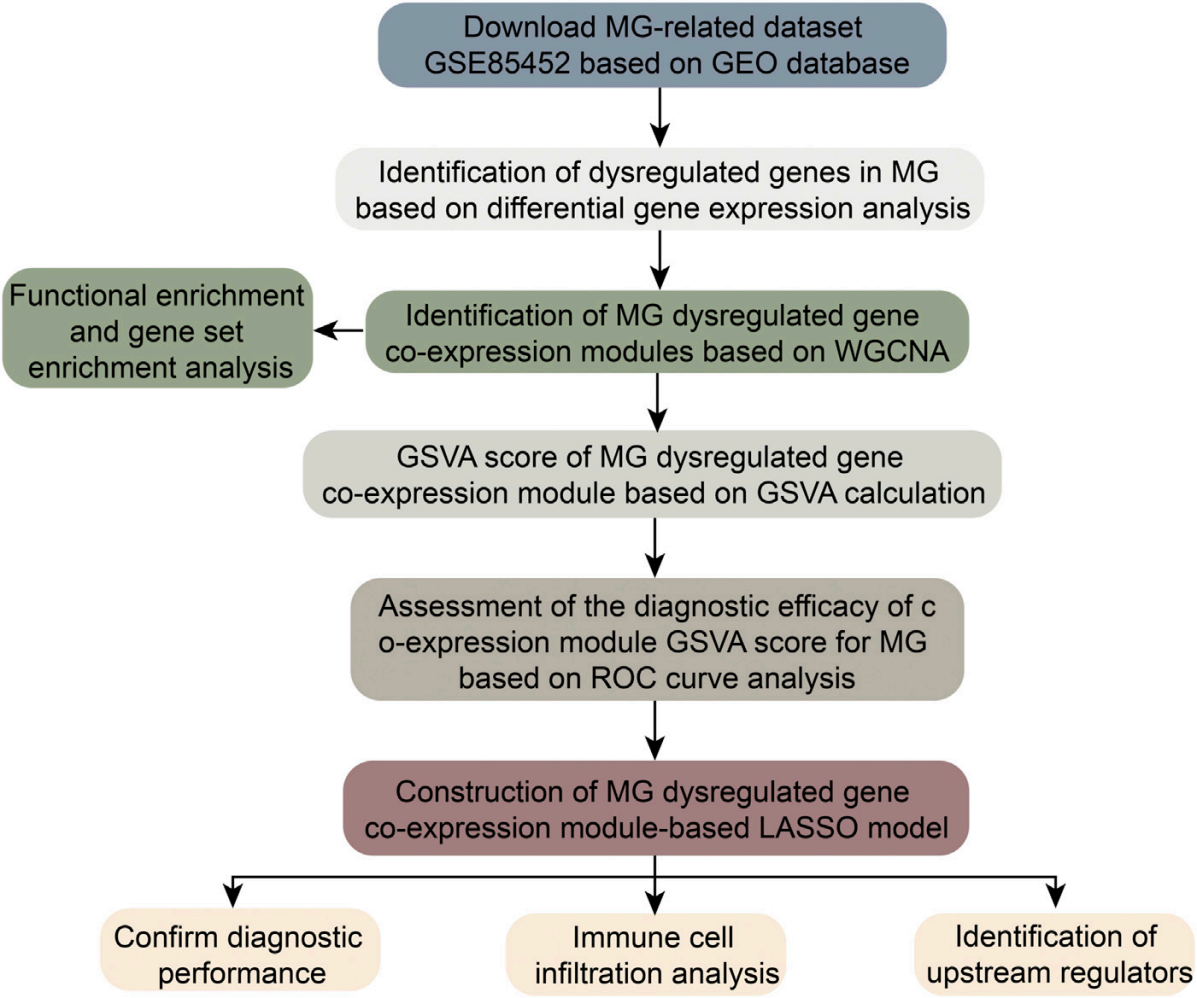
Flowchart of this work.

**FIGURE 2:**
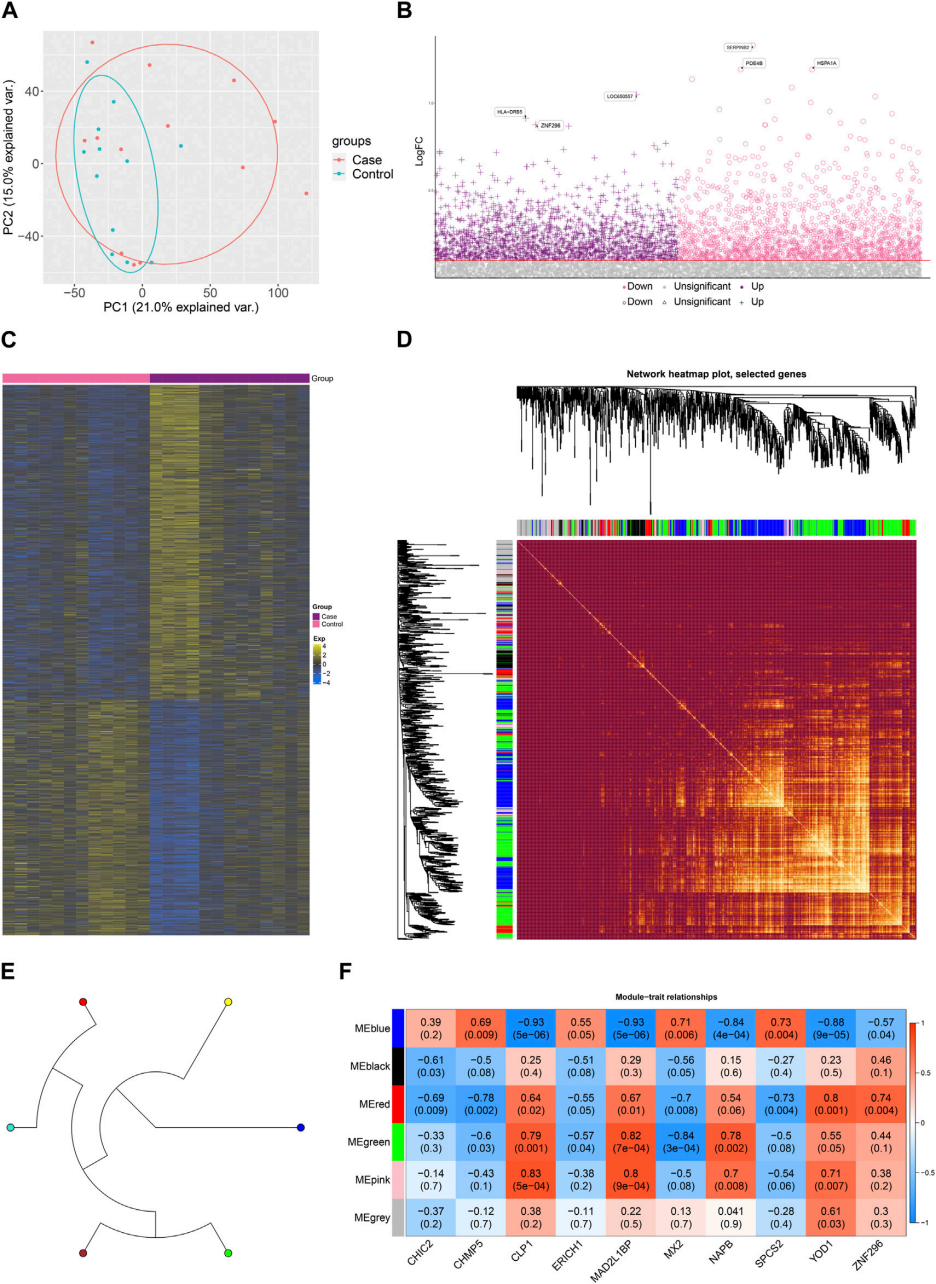
Dysregulated gene co-expression modules characterize the global regulatory pattern of myasthenia gravis. **(A)**. Principal component analysis (PCA) plots showing significant differences between disease and control. **(B)** Manhattan plots showing differential expression of Case-Control. **(C)** Heat map showing expression of dysregulated genes in Case-Control groupings. **(D)**. Module ring tree plots showing neighboring relationships between dysregulated gene co-expression modules. **(E)** Module heat map showing gene members of dysregulated gene co-expression modules. **(F)** Module correlation plot showing the expression correlation of gene co-expression modules with Top10 significantly dysregulated genes.

As shown in Figure 1A, gene expression data from PBMC samples of 13 MG patients and 12 healthy controls were analyzed in this study. The bias of sequencing data due to gene length, sequencing volume and other factors was removed by normalization. Principal component analysis (PCA) scatter plots showed good discrimination between different samples (Figure 2A). DEGs between MG and control were identified by differential gene expression analysis, including 861 upregulated DEGs and 643 downregulated DEGs (Figure 2B, Supplementary Table S1). The heat map showed that these dysregulated genes could significantly distinguish between MG and control samples (Figure 2C).

Subsequently, to further explore the relationship between these dysregulated genes and MG, this study screened these genes for WGCNA. to construct a scale-free network, we set the soft threshold power β to 16, and DEGs with similar expression patterns were co-classified into six co-expression modules. The module ring tree diagram demonstrates the neighbor-joining relationships among the dysregulated gene co-expression modules (Figure 2D). In addition, the module heat map further demonstrates the co-expression of MG dysregulated genes in different modules (Figure 2E). The expression correlations of some significantly dysregulated genes were demonstrated in the module correlation plots (Figure 2F), which may be closely related to MG development.

### Biological functions and signaling pathways significantly involved in myasthenia gravis dysregulated gene co-expression modules

**FIGURE 3:**
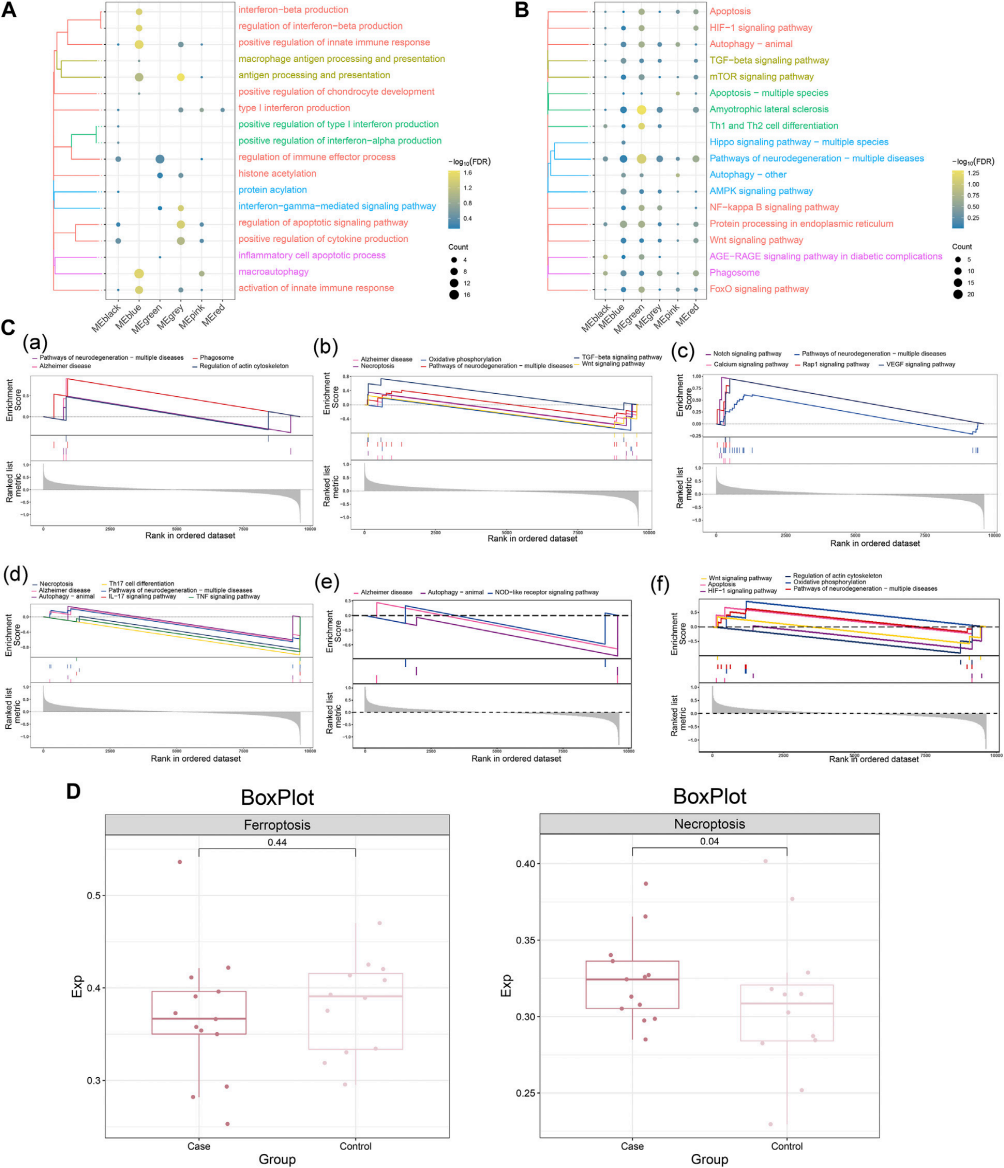
Biological functions and signaling pathways significantly involved in myasthenia gravis dysregulated gene co-expression modules. **(A)** Clustered bubble plots showing the biological functions significantly regulated by MG dysregulated gene co-expression modules (listed as different modules). **(B)** Clustered bubble plots showing the signaling pathways significantly regulated by MG dysregulated gene co-expression modules (listed as different modules). **(C)** Comprehensive GSEA diagram showing signaling pathways significantly activated/repressed by MG dysregulated gene co-expression modules, **(a)** black module, **(b)** blue module, **(c)** green module, **(d)** gray module, **(e)** pink module, **(f)** red module. **(D)** Scoring of ferroptosis and necroptosis in MG and control samples.

To further investigate the biological functions and signaling pathways significantly involved in MG dysregulated gene co-expression modules, enrichment analysis of these genes was performed. The results showed that these genes are significantly involved in the biological processes of positive regulation of innate immune response, regulation of interferon-beta production and positive regulation of cytokine production and KEGG pathways such as neurodegeneration-multiple diseases pathway, Th1 and Th2 cell differentiation, TGF -beta signaling pathway and mTOR signaling pathway. (Figures 3A, B). In addition, GSEA further confirmed the activation or inhibition of KEGG signaling pathways in different co-expression modules (Figure 3C), suggesting that these pathways may play an important role in the development of MG. In addition to this, we compared the scores of ferroptosis and necroptosis in MG and control samples and found that the scores of necroptosis were higher in the MG group, while the scores of ferroptosis were not significant (Figure 3D).

### Myasthenia gravis dysregulated gene co-expression module-based clinical model has significant diagnostic efficacy

**FIGURE 4:**
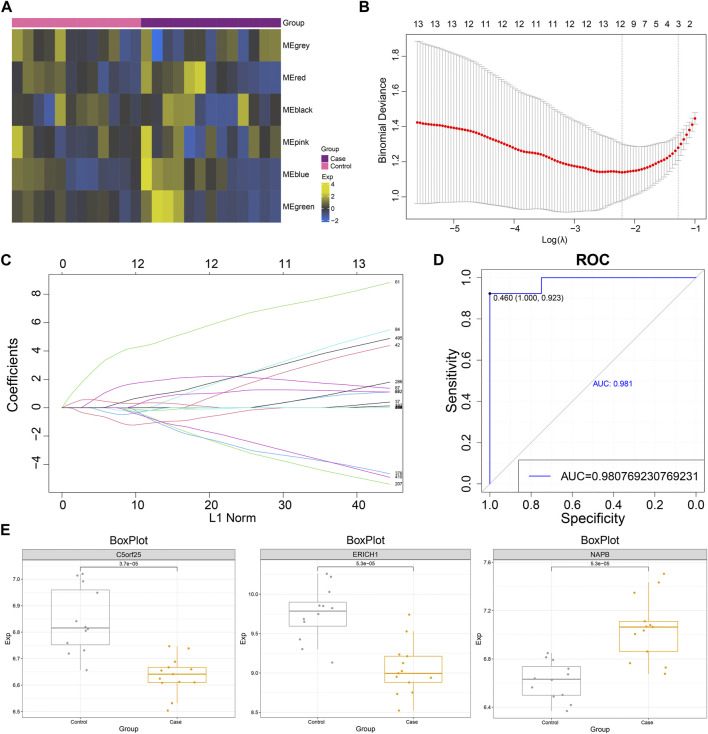
The myasthenia gravis dysregulated gene co-expression module-based clinical model has significant diagnostic efficacy. **(A)** Heat map demonstrating significant enrichment of MG dysregulated gene co-expression module gene (GSVA) scores in Case-Control. **(B)** Lambda plot demonstrating model performance of the set of MG dysregulated gene co-expression module functional genes with diagnostic efficacy at different Lambda (single factor significant genes were selected for Lasso modeling). **(C)** Lasso model Plot showing the model confidence of the set of functional genes with diagnostic efficacy of MG dysregulated gene co-expression module at different log (Lambda). **(D)** ROC curve showing the ROC curve of MG dysregulated gene co-expression module-based model. **(E)** Box plot showing the expression level of model genes.

The GSVA scores of MG dysregulated gene co-expression modules were calculated based on the GSVA method (Figure 4A), and the diagnostic efficacy of GSVA scores of different modules for MG was identified using ROC analysis. The results showed that the Green module had the best diagnostic efficacy for MG (AUC = 0.584, Supplementary Figure S1). Subsequently, the MG dysregulated gene co-expression module-based clinical model was further constructed using the LASSO method, and three characteristic genes with non-zero regression coefficients were obtained (lambda.min = 0.110, Figures 4B,C). ROC curve analysis showed that the MG dysregulated gene co-expression module-based model showed excellent diagnostic efficacy for MG (AUC = 0.981, Figure 4D), and some of these genes, such as NAPB, showed significantly high expression in MG, while C5orf25 and ERICH1 showed significantly low expression in MG (Figure 4E). In addition, the correlation between NAPB, C5orf25 and ERICH1 genes and ferroptosis and necroptosis was analyzed and the results were shown in Supplementary Table S2, ERICH1 was negatively associated with necroptosis.

### Co-expression module reprograms the immune microenvironment of myasthenia gravis

**FIGURE 5:**
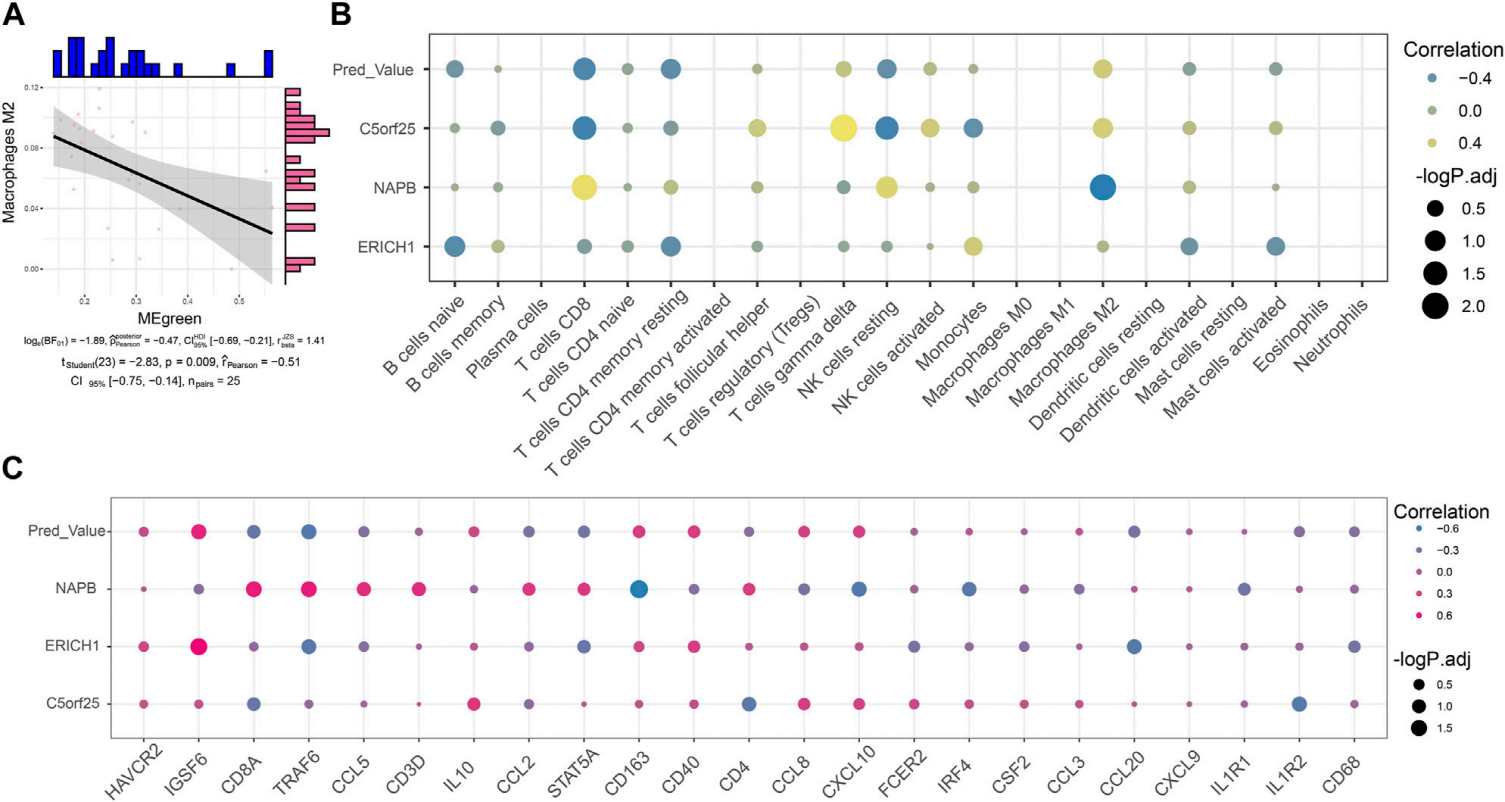
Co-expression modules reprogram the immune microenvironment of myasthenia gravis. **(A)** Series of correlation scatter plots showing expression correlation of immune cell infiltration abundance with diagnostic potency of MG dysregulated gene co-expression module scores. **(B)** Bubble plots showing correlation of MG dysregulated gene co-expression module-based Lasso model genes with immune cell infiltration abundance. **(C)** Bubble plots showing correlation of MG dysregulated gene co-expression module-based Lasso model genes correlated with immune checkpoint-associated genes and tertiary lymphoid structural marker genes.

By immune infiltration analysis, this study explored the level of immune cell infiltration in Control and MG samples. Correlation analysis showed a significant negative correlation between the Green module score and the infiltration abundance of Macrophages M2 cells, suggesting that the expression of these module genes may inhibit the infiltration of the corresponding immune cells (Figure 5A). Notably, MG dysregulated gene co-expression module scores as well as scored genes showed significant correlation with the abundance of some immune cells, immune checkpoint genes and tertiary lymphoid structural marker genes (Figures 5B, C), suggesting that these module genes may be indirectly involved in reprogramming the MG immune microenvironment by promoting the infiltration of immune cells, or regulating the expression of immune-related genes.

### Upstream regulators of dysregulated gene co-expression modules

**FIGURE 6:**
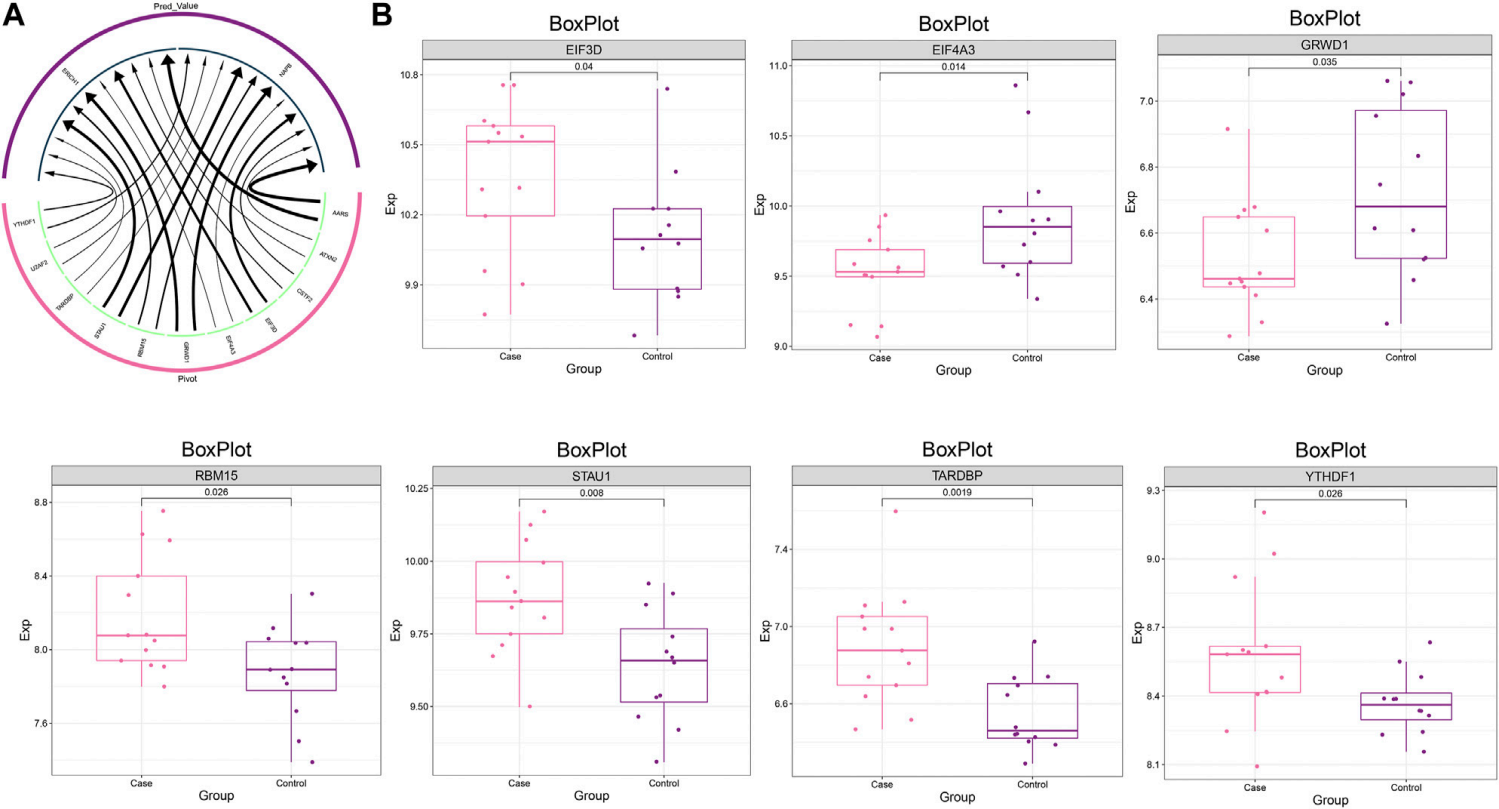
Upstream regulators of the dysregulated gene co-expression module. **(A)** Circular network plot demonstrating the regulatory effect of RBP on MG dysregulated gene co-expression module-based Lasso model genes. **(B)** Box plot demonstrating the expression level of RBPs.

To construct a global regulatory network for the MG dysregulated gene co-expression module-based model, we further explored the upstream regulators of these genes. The upstream regulators regulating this model gene set, including RBPs such as YTHDF1, U2AF2, TARDBP, STAU1, were identified by Pivot analysis (Figure 6A), where EIF3D, RBM15, STAU1, TARDBP and YTHDF1 showed significant high expression in MG samples (Figure 6B).

## Discussion

The clear pathogenesis of MG is still unknown, and due to its heterogeneous and complex pathogenesis, there are no effective treatment options for MG patients (Sanders et al., 2016). The present study is based on the mRNA expression profile of MG from the GEO database. In this study, we identified MG-related DEGs for WGCNA based on the mRNA expression profile of MG in the GEO database, and searched for the most relevant modules to construct a scoring clinical model, which can adequately ensure the interaction between genes. Then, the diagnostic efficacy of the scoring clinical model for MG was determined. In addition, few established immune-related gene profiles were combined with conventional prognostic models to optimize routine clinical practice. They are not very effective as a direct guide to clinical workup. To remedy these shortcomings, we further explored the immune microenvironment of MG based on clinical models of MG scoring. These findings strongly suggest a great potential role for the MG dysregulated gene co-expression module-based model in MG obtained in this study.

WGCNA makes strongly correlated genes strongly correlated after power function treatment, therefore, the construction of WGCNA network helps to identify and screen important modules and key genes associated with specific clinical phenotypes (Langfelder and Horvath, 2008). In this study, WGCNA analysis was performed on RNA-seq datasets downloaded from the GEO database, and DEGs were calculated separately between MG patients and healthy controls, yielding a total of 1504 DEGs as the dataset for subsequent co-expression network analysis to prevent high correlations for genes that were not significantly different. Notably, the co-expression network analysis identified and clustered into six co-expression modules, and the correlation analysis of genes with significantly dysregulated genes was performed for each module, and the Top10 pivotal genes contained in each module were screened for strong interaction with MG, respectively.

Among the different modules, genes were found to be mainly enriched in Pathways of neurodegeneration - multiple diseases, Alzheimer disease, Th1 and Th2 cell differentiation, Regulation of actin cytoskeleton, Oxidative phosphorylation, Necroptosis, TGF-beta signaling pathway, Wnt signaling pathway and other KEGG signaling pathways. Notably, each module was significantly enriched in Pathways of neurodegeneration - multiple diseases. In addition, functional enrichment analysis revealed that module genes were mainly enriched in positive regulation of innate immune response, regulation of interferon-beta production, positive regulation of cytokine production, activation of innate immune response This suggests that the main cause of MG development is abnormalities in interferon and immune pathways. In the course of MG, abnormal antigen processing and presentation may contribute to the onset and progression of the disease (Xu et al., 2021). In addition, it has been found that imbalance of various helper T cells (including Th1, Th2, Th17, Th22 and follicular helper T (TFH) cells in MG is associated with immune disorders, suggesting that the balance of Th cells and their cytokines in MG patients is related to the clinical status or severity of MG disease (Wang et al., 2019). It has also been shown that oxidative stress and low antioxidant status play a major role in the pathogenesis of inflammatory and autoimmune diseases, and that MG patients with low antioxidant status have active oxidative processes (Yang et al., 2016; Adamczyk-Sowa et al., 2017). In addition to this, studies have confirmed that AChR-MG may be an acquired interferon disease (Payet et al., 2022). The results of GO and KEGG analysis in this study also suggest that MG dysregulated genes are mainly enriched in interferon and immune-related processes.

To date, there are no studies on the NAPB, C5orf25, and ERICH1 genes in PBMCs in MG. The N-ethylmaleimide-sensitive accessory protein beta (NAPB) gene is associated with brain development as well as brain development in neurological disorders, such as various severe early onset epilepsy (Conroy et al., 2016; Zhao et al., 2021). In addition, NAPB has been shown to act as a pivotal gene in Alzheimer’s disease and to be involved in the pathogenesis of Alzheimer’s disease (Zhang et al., 2020). CAPN3 has been reported to have multiple muscle cell functions and mutations in this protease cause limb-girdle muscular dystrophy type 2A (Ono et al., 2013). C5orf25 is a novel CAPN3-binding protein that regulates the protease activity of CAPN3 and has the potential to act as a scaffolding protein (Ono et al., 2013). While ERICH1 has been reported to be associated with the risk of multiple sclerosis (MS) (Maltby et al., 2017), MS and MG are two uncommon neurological problems, both of which can affect the nervous system.

Immune cell infiltration analysis showed a significant negative correlation between the infiltration abundance of green module Macrophages M2 cells. Macrophages the cause of the pathogenesis of some human neuroimmune diseases, mainly MS, optic neuromyelitis optica (NMO), MG and Guillain-Barré syndrome (GBS) (Fan et al., 2016).

However, the present study still has limitations, as the sample size of the public database is too small, which may lead to the omission of pivotal genes. In addition, the results of this analysis were obtained exclusively by bioinformatics and failed to experimentally validate the expression of the obtained biomarkers at protein and RNA levels, and further experimental validation is proposed in the future.

## Conclusion

The results of this study showed that a gene-based clinical model consisting of NAPB, C5orf25 and ERICH1 showed high diagnostic ability for MG (AUC = 0.981), and this model developed can be used as a diagnostic indicator for MG, which is crucial for subsequent clinical treatment and improvement of disease prognosis.

## Data Availability

The datasets presented in this study can be found in online repositories. The names of the repository/repositories and accession number(s) can be found in the article/Supplementary Material.

## Abbreviations

MG, myasthenia gravis; GEO, gene expression omnibus; DEGs, Differentially expressed genes; WGCNA, Weighted gene co-expression network analysis; GSVA, Gene set variance analysis; LASSO, Least absolute shrinkage and selection operator; AChR, acetylcholine receptor; MuSK, muscle-specific kinase; PBMCs, peripheral blood mononuclear cells; GO, Gene Ontology; KEGG, kyoto encyclopedia of genes and genomes; GSEA, gene set enrichment analysis; MsigDB, molecular signature database; ROC, recipient operating characteristic; AUC, area under the curve; RBPs, RNA binding proteins; PCA, principal component analysis; NAPB, N-ethylmaleimide-sensitive accessory protein beta; MS, multiple sclerosis; NMO, neuromyelitis optica; GBS, guillain-barré syndrome.
