# Supplementary figures and images for: Transcriptional landscape of myasthenia gravis revealed by weighted gene coexpression network analysis
Source: Front Genet. 2023 Mar 27;14:1106359. doi: 10.3389/fgene.2023.1106359 (PMC10083720; doi:10.3389/fgene.2023.1106359)

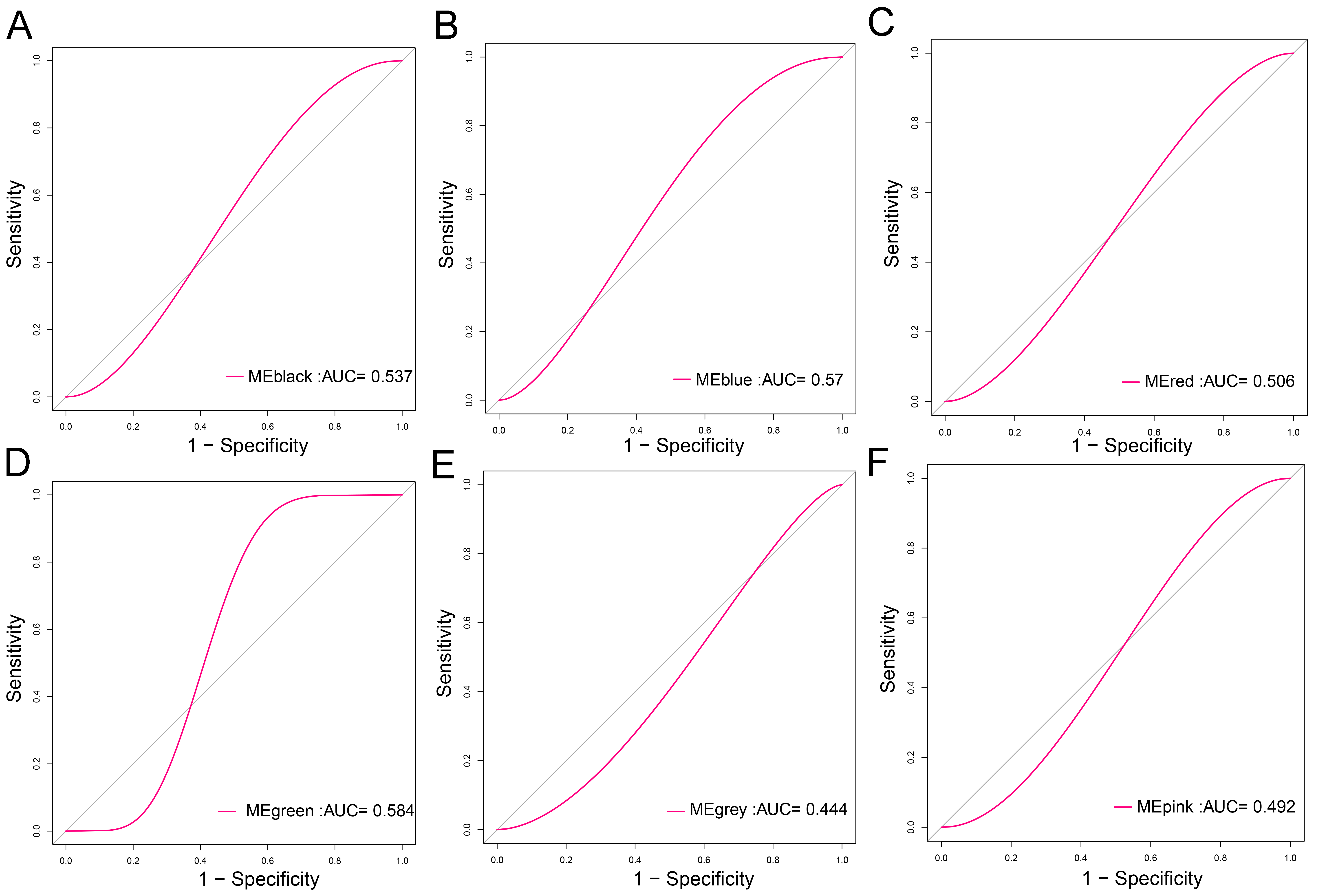

Supplement: Supplementary file 2 [file Image1.TIF]
